# Salivary Alterations of Myeloperoxidase in Patients with Systemic Diseases: A Systematic Review

**DOI:** 10.3390/ijms241512078

**Published:** 2023-07-28

**Authors:** Kacper Nijakowski, Jakub Jankowski, Dawid Gruszczyński, Anna Surdacka

**Affiliations:** 1 Department of Conservative Dentistry and Endodontics, Poznan University of Medical Sciences, 60-812 Poznan, Poland; annasurd@ump.edu.pl; 2 Student’s Scientific Group in Department of Conservative Dentistry and Endodontics, Poznan University of Medical Sciences, 60-812 Poznan, Poland; jjankowski41@wp.pl (J.J.); dawid.j.gruszczynski@gmail.com (D.G.)

**Keywords:** myeloperoxidase, saliva, systemic disease, coronary artery disease, inflammatory bowel disease, asthma, obstructive sleep apnoea, COVID-19, haematopoietic stem cell transplantation, antioxidant

## Abstract

Salivary myeloperoxidase (MPO) is a key mediator of the oral immune system, acting as an enzyme that utilises H_2_O_2_ to generate molecules with high bactericidal activity. While MPO determination in plasma is quite common, the use of saliva is still rare. Our systematic review was designed to answer the question “Are salivary levels of myeloperoxidase altered in patients with systemic diseases?”. Following the inclusion and exclusion criteria, we included twenty-six studies. Altered MPO levels in saliva were most commonly found in patients with cardiovascular and gastrointestinal diseases. Most studies concerned unstimulated whole saliva, and only a few of them stimulated, mainly by chewing paraffin. Enzyme-linked immunosorbent assay (ELISA) was the most common method for determination of MPO concentrations in saliva. Increased salivary MPO levels were more often observed for inflammatory diseases, except patients with inflammatory bowel diseases who were eligible for biologic therapy. In conclusion, MPO could be altered in the saliva of patients with systematic diseases, especially cardiovascular or gastrointestinal diseases. However, further investigations are recommended to validate these outcomes.

## 1. Introduction

Myeloperoxidase (MPO) is a heme-containing enzyme (E.C.1.11.1.7) found primarily in neutrophils that catalyses the formation of reactive oxygen and nitrogen species with potent antimicrobial activity [1]. Typically, MPO utilises H_2_O_2_ generated by NADPH oxidases, xanthine oxidase, or nitric oxide synthase to produce hypochlorous acid with strong bactericidal properties [2]. In addition, by consuming H_2_O_2_, MPO protects the host from the toxicity of bacterial H_2_O_2_. For these reasons, MPO is viewed as a key component of innate immunity and an inflammatory mediator [3,4].

On the other hand, however, uncontrolled MPO activity can lead to excessive oxidant production, oxidative modifications of macromolecules, and tissue damage. In this respect, MPO-derived oxidants have been implicated in the pathogenesis of many diseases characterised by chronic inflammation, including atherosclerosis and cardiovascular disease, renal, liver and gastrointestinal diseases, cancer, rheumatoid arthritis, and neurodegenerative diseases [5]. Therefore, it has been proposed that MPO may serve as a biomarker to assess the risk of developing these conditions [6,7].

While the measurement of MPO in plasma is recognised in biomedical research, the use of saliva for this purpose is rare. In turn, the use of saliva as a biological fluid in the diagnosis of systemic diseases (e.g., gastrointestinal diseases, oncological diseases) is already better understood [8,9,10].

It is known that MPO levels in saliva depends on two main reasons: the natural migration of neutrophils into saliva and oral fluid, and as a result of an inflammatory response of the mucous membranes in diseases of the oral cavity. We hypothesised that MPO levels in saliva may reflect systemic changes in the body, just as oral health has a bidirectional effect on general health. Therefore, our systematic review was designed to answer the question “Are salivary levels of myeloperoxidase altered in patients with systemic diseases?”. By the term “systemic diseases”, we mean disorders that can affect a few organs and tissues or even the whole body, such as cardiovascular diseases, respiratory diseases, gastrointestinal diseases, haematological diseases, autoimmune diseases, endocrine diseases, etc., without presenting non-specific oral manifestations.

## 2. Materials and Methods

### 2.1. Search Strategy and Data Extraction

The present systematic review was conducted up to 5 May 2023, according to the Preferred Reporting Items for Systematic Reviews and Meta-Analyses (PRISMA) statement guidelines [11], using the databases PubMed, Scopus, and Web of Science. The search queries included:

- for PubMed: (myeloperoxidase AND saliva) AND (disease OR disorder OR syndrome OR therapy)
- for Scopus: TITLE-ABS-KEY ((myeloperoxidase AND saliva) AND (disease OR disorder OR syndrome OR therapy))
- for Web of Science: TS = ((myeloperoxidase AND saliva) AND (disease OR disorder OR syndrome OR therapy)).

Records were screened by the title, abstract, and full text by two independent investigators. Studies included in this review matched all the predefined criteria according to PI(E)COS (“Population”, “Intervention”/”Exposure”, “Comparison”, “Outcomes”, and “Study design”), as reported in Table 1. We did not exclude studies that simultaneously reported data for patients with a given systemic disease depending on different periodontal status—it was crucial to provide MPO levels for periodontally healthy individuals. A detailed search flowchart is presented in the Results section. The study protocol was registered in the international prospective register of systematic reviews PROSPERO (CRD42023424607).

### 2.2. Quality Assessment and Critical Appraisal for the Systematic Review of Included Studies

The risk of bias in each individual study was assessed according to the “Study Quality Assessment Tool” issued by the National Heart, Lung, and Blood Institute within the National Institute of Health [12]. These questionnaires were answered simultaneously by two independent investigators, and any disagreements were resolved by discussion between them. The summarised quality assessment is reported in the Results section. 

The level of evidence was assessed using the classification of the Oxford Centre for Evidence-Based Medicine levels for diagnosis [13].

## 3. Results

**Figure 1:**
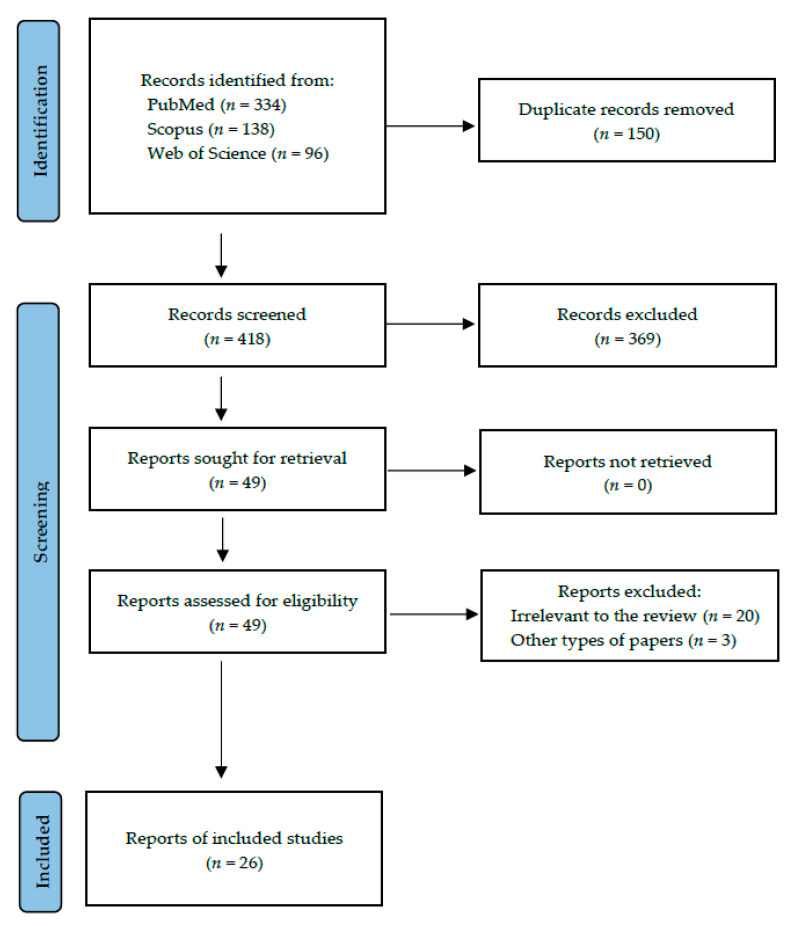
PRISMA flow diagram presenting search strategy.

Following the search criteria, our systematic review included twenty-six studies, demonstrating data collected in thirteen different countries from a total of 1812 participants with diagnosed systemic diseases. Figure 1 reports the detailed selection strategy of the records. The inclusion and exclusion criteria are presented in the Materials and Methods section.

In Table 2, we collected data about the general characteristics of each eligible study, such as year of publication and setting, which involved participants and their diagnosis, inclusion and exclusion criteria, and smoking status. The majority of studies came from Europe (most from Finland—six studies). Altered MPO levels in saliva were most commonly observed in patients with cardiovascular and gastrointestinal diseases. Table 3 shows the detailed characteristics considering types of saliva and methods of their collection, centrifugation and storing conditions, laboratory MPO determination method, and other biomarkers potentially altered in saliva. Most studies concerned resting mixed saliva, collected as described by Navazesh [14], and only a few of them stimulated, mainly by chewing paraffin pieces. In saliva, MPO concentrations were most commonly determined by enzyme-linked immunosorbent assays (ELISA) and activity using the modified method by Mansson-Rahemtulla et al. [15]. Only two studies evaluated MPO activity rather than its concentration. Included studies involved different conditions of processing saliva—most often it was centrifuged and frozen at −20 to −80 °C until laboratory analysis. In addition, the statistically significant outcomes about salivary MPO levels from included studies (which reported accurate values, not only in the plots) are presented in Table 4. In general, it is impossible to draw consistent observations as to the upward or downward trends of MPO levels in systemic diseases. However, elevated MPO concentrations were more commonly found for inflammatory diseases, with the main interesting exception being patients with inflammatory bowel diseases who were eligible for biologic therapy.

**Figure 2:**
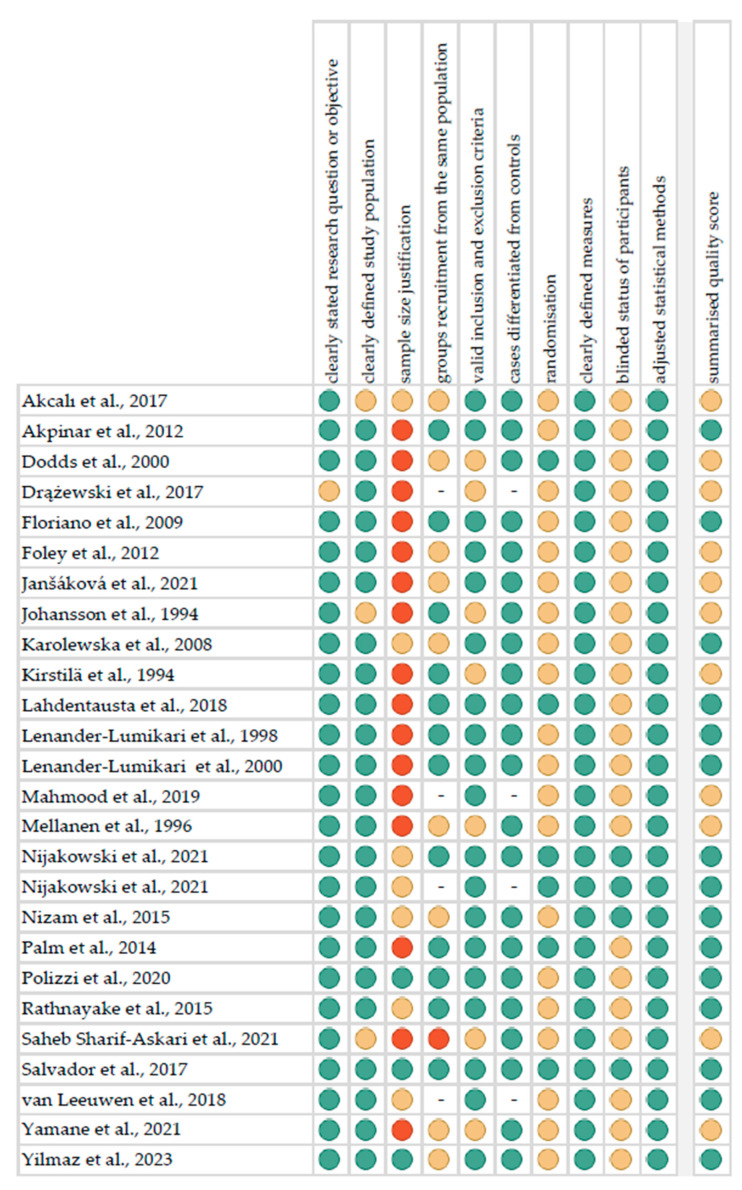
Quality assessment, including the main potential risk of bias (risk level: green—low, yellow—unspecified, red—high; quality score: green—good, yellow—intermediate, red—poor) [16,17,18,19,20,21,22,23,24,25,26,27,28,29,30,31,32,33,34,35,36,37,38,39,40,41].

Figure 2 reports the summarised quality assessment, according to the “Study Quality Assessment Tool” issued by the National Heart, Lung, and Blood Institute within the National Institute of Health [12]. The most frequently encountered risks of bias were the absence of data regarding blinding (twenty-two studies), randomisation (twenty studies), and sample size justification (sixteen studies). Critical appraisal was summarised by adding up the points for each criterion of potential risk (points: 1—low, 0.5—unspecified, 0—high). Fifteen studies (57.7%) were classified as having “good” quality (≥80% total score) and eleven (42.3%) were classified as “intermediate” (≥60% total score).

Most of the included studies had the third or fourth level of evidence (case-control studies), according to the five-graded scale the classification of the Oxford Centre for Evidence-Based Medicine levels for diagnosis [13]. Only three studies demonstrated the prospective cohort design.

## 4. Discussion

Our systematic review discusses previous studies on the diagnostic use of salivary MPO levels in patients with systemic diseases, such as cardiovascular diseases, respiratory disorders, gastrointestinal diseases, haematological disorders, infectious and immunological disorders, autoimmunological disorders, and others. Due to the heterogeneity of the included studies, this section was deliberately divided into disease subgroups.

### 4.1. Cardiovascular Diseases

The levels of neutrophil-associated plasma proteins, such as MPO and matrix metalloproteinase 9 (MMP-9), could predict the risk of cardiovascular events related to the severity of atherosclerosis [42,43]. In patients with cardiovascular diseases, neutrophils are more prone to release these mediators than neutrophils from healthy controls [44]. MPO and MMP-9 are related to such processes as inflammation, tissue damage, and tissue remodelling in individuals with myocardial infarction [45].

MPO and MPO-derived oxidants can contribute to atherosclerosis by foam cell formation and endothelial dysfunction demonstrated by increased endothelial cell permeability and apoptosis [46]. Also, they activate latent matrix metalloproteinases (MMPs) and tissue factor expression, promoting the vulnerable plaque [47]. Unstable or ruptured plaque may be caused by MPO-induced superficial plaque erosion and increased susceptibility to thrombus formation [48]. The MPO-mediated modification of cholesterol efflux from lipid-laden cells attenuates the anti-atherogenic properties of high-density lipoproteins [49,50]. In contrast, MPO is involved in the formation of atherogenic oxidised low-density lipoproteins, which lead to the development of atherosclerotic plaques with an enlarged lipid core pressing on the fibrous cap matrix [51].

The study by Polizzi et al. [35] presented that patients with coronary heart disease demonstrated significantly elevated salivary and plasma levels of MPO, regardless of periodontal status. Based on the multivariate regression analysis, in these patients, salivary MPO concentrations could be predicted by CRP and total cholesterol levels. In turn, Lahdentausta et al. [26] suggested that MPO may be a reliable marker for both acute coronary syndrome (ACS) and periodontal disease, but this depends on the type of biological material. Salivary levels were useful for diagnosing periodontitis but not for diagnosing ACS. The opposite finding was true for serum MPO.

Moreover, Mahmood et al. [29] observed a significant increase in salivary MPO levels after exercise in patients with coronary artery disease. However, the protein-adjusted levels did not differ significantly from those at rest. MPO levels in saliva were 4-fold higher than in plasma and did not correlate with each other. 

Interestingly, Foley et al. [21] evaluated if salivary biomarkers could demonstrate utility for identifying myocardial necrosis. Salivary MPO was characterised by a downward trend with a significantly lower level than the baseline at 48 h after the alcohol septal ablation in patients with hypertrophic cardiomyopathy.

Rathnayake et al. [36] investigated salivary MPO levels in patients with myocardial infarction (MI). After adjusting for gingival status and smoking habits, MPO concentrations in saliva were significantly lower than in the control group. Males demonstrated significantly higher MPO levels compared with females. Also, clinical signs of periodontal inflammation positively correlated with salivary MPO concentrations.

Similarly, Floriano et al. [20] investigated the utility of saliva for identifying alternative biomarkers of acute myocardial infarction (AMI). In contrast, MPO levels were increased in AMI patients compared to controls in both saliva and serum, almost 2- and 3-fold, respectively. The salivary panel of MPO, myoglobin, and C-reactive protein presented a significant diagnostic capability for AMI (AUC = 0.85), which increased when an electrocardiogram (ECG) was added (AUC = 0.94). At that time, the screening value was comparable to the panel of troponin-I, creatine kinase-MB, myoglobin, and brain natriuretic peptide (AUC = 0.98), and was significantly higher than ECG itself.

In addition, Palm et al. [34] determined that patients with ischemic stroke had significantly lower salivary MPO concentrations than the control subjects. Similar findings were presented for serum MPO concentrations. After adjustment, differences remained significant.

### 4.2. Respiratory Disorders

The role of neutrophils in airway inflammation in the course of asthma is confirmed. In children and adults with severe asthma, blood or sputum MPO is increased, reflecting elevated neutrophil activity [52]. Also, the catalytic activity of MPO is modulated by plasma nitric oxide released during chronic inflammation in patients with bronchial asthma [53].

In 1998, Lenander-Lumikari et al. [27] observed decreased stimulated salivary flow rates and increased MPO concentrations in asthmatic adults compared with non-asthmatic ones. They speculated that higher MPO levels might be associated with a higher degree of periodontal inflammation (described by Periodontal Status Index) in asthmatics.

Obstructive sleep apnoea (OSA), presenting as upper airways collapse during sleep, leads to intermittent hypoxia. Therefore, oropharyngeal inflammation is associated with increased levels of proinflammatory cytokines and reactive oxygen species (ROS) [54,55]. During inflammation involving both upper and lower airways, the release of neutrophilic enzymes, such as MPO, MMPs, and neutrophil elastase, is elevated [56]. Moreover, MPO is considered as a mutual contributor to the higher incidence of OSA and cardiovascular diseases [57].

In the study by Akpinar et al. [17], patients with OSA demonstrated significantly higher levels of MPO in saliva compared to healthy controls. Serum differences were at the borderline of statistical significance. Salivary MPO levels positively correlated with the Apnoea-Hypopnea Index (AHI), the oxygen desaturation index, and sleep efficiency. The authors suggest that salivary MPO could be a useful oropharyngeal inflammatory marker in OSA patients. However, in the study by Nizam et al. [33], patients with mild-moderate and severe obstructive sleep apnoea syndrome demonstrated lower levels of MPO in saliva and serum compared with the healthy controls, but these differences were not significant.

### 4.3. Gastrointestinal Diseases

In active inflammatory bowel diseases, the mucosal barrier is injured by intestinal inflammatory and oxidative processes caused by enhanced neutrophil infiltration [58,59]. The increased production of proinflammatory cytokines and ROS is modulated by neutrophil recruitment and accumulation in the gastrointestinal wall [60]. The intestinal mucosal integrity is ensured by maintaining a balance between ROS and antioxidants, including MPO, which is responsible for the formation of neutrophil extracellular traps [61,62]. Importantly, MPO seems to be a therapeutic target for protecting colon mucosa from inflammatory damage [63].

In our previous study, patients with inflammatory bowel diseases eligible for biologic therapy had significantly reduced MPO levels in the saliva of patients with ulcerative colitis (UC) compared to patients with Crohn’s disease (CD) and healthy controls. Based on the ROC analysis, the lower salivary MPO concentrations could be a significant predictor for the differential diagnosis between CD and UC [31]. Furthermore, as a result of an effective response to biologic treatment, only patients with UC demonstrated MPO levels significantly increased to those comparable to healthy subjects [32].

Our most recent cross-sectional study in Polish patients with IBD [64] found that lowered MPO concentrations in saliva could be a predictor for the non-invasive diagnosis of clinically active UC, and was significantly correlated with the endoscopic severity in this group. Also, salivary MPO in patients treated biologically and without steroid therapy demonstrated significant correlations with selected blood parameters, reflecting inflammatory status (such as CRP or white blood cells). Our findings suggest that MPO levels in saliva could be used to monitor IBD activity and treatment effectiveness.

In contrast, Janšáková et al. [22] found only a slight increase in salivary MPO levels in patients with CD and orofacial granulomatosis (OFG) compared with the control group.

In 2000, Lenander-Lumikari et al. [28] presented that salivary peroxidase and MPO activities were significantly elevated in patients with coeliac disease compared with healthy subjects. In turn, the gluten challenge resulted in a decrease in MPO activity in these patients. However, no differences in stimulated saliva flow rates were found. A non-gluten diet, including long-chain omega-3 fatty acids, flavonoids and carotenoids, can modulate the expression and production of oxidative and inflammatory mediators, preserving intestinal barrier integrity [65]. Inflammation caused by an imbalance between oxidant and antioxidant markers (including MPO) may stimulate DNA damage [66].

Chronic protein–energy malnutrition is associated with the permanent disruptions of salivary glands, which lead to decreased protein production [67]. In the study by Johansson et al. [23], no differences in MPO concentrations were observed between the saliva samples from Indian children with chronic protein–energy malnutrition and the control group.

### 4.4. Haematological Disorders

The suppression of salivary defence mechanisms appears after the chemotherapy introduction, not haematological disorders themselves [68]. The cytostatic treatment causes significant decreases in saliva secretion rates and the lack of peripheral blood granulocytes, leading to an extremely lowered count of neutrophils in saliva and, subsequently, decreased MPO activity [69,70]. However, the decreased MPO-dependent antimicrobial defence may be compensated by increased lactoferrin release in saliva [71].

The oral neutrophils reach normal counts in saliva even before peripheral blood [72]. Altered MPO activity can result in excessive accumulation of H_2_O_2_, which is responsible for oral tissue damage. Patients demonstrating oral mucositis with ulcerative lesions seem to favour the neutrophilic infiltration [73]. During oral mucositis, blood proteins pass into saliva due to leakage in the integrity of the oral mucosa [74].

In children with newly diagnosed acute leukaemia, Karolewska et al. [24] assessed the changes in the activity of salivary antibacterial factors in the course of leukaemia, depending on the oral clinical findings. Patients with aplasia demonstrated significantly lower levels of MPO and peroxidase in saliva. In addition, the significantly decreased salivary activities of MPO and peroxidase were presented in leukaemia children with mucositis compared to those without mucositis.

In the study by van Leeuwen et al. [39], the salivary MPO demonstrated the fluctuating trends in multiple myeloma patients treated with high-dose melphalan and autologous haematopoietic stem cell transplantation (HSCT). In unstimulated saliva, the lowest MPO levels were measured one week after transplantation. Also, MPO concentrations were similarly low on the day of transplantation, with an increase on the fourth and eleventh postoperative days. For MPO in stimulated saliva, the changes did not show statistical significance. At the same time, decreases in the secretion of both resting and stimulated saliva were observed.

Moreover, Salvador et al. [38] evaluated the effect of photobiomodulation (PBM) therapy on the reducing severity of oral mucositis in patients undergoing HSCT. In the study group, patients received PBM applications every day until the seventh post-transplant day, and in the control group, only the oral hygiene guidelines were applied. In both groups, salivary MPO levels significantly decreased one week after transplantation.

### 4.5. Infectious and Immunological Disorders

The study by Mellanen et al. [30] showed elevated levels of MPO in the saliva of HIV-seropositive patients. The authors speculated that this increase might be related to the severity of periodontal disease. In the study by Kirstilä et al. [25], patients with common variable immunodeficiency did not differ from the control subjects concerning MPO levels in saliva. However, total salivary peroxidase activity was significantly elevated in immunodeficient patients. Unexpectedly, MPO can also be present in human lymphocytes. The increased MPO in CD4(+) T lymphocytes from chronic HIV infection is found [75]. In chronic HIV, mitochondrial dysfunction can be induced by MPO, leading to the vicious cycle of mitochondrial damage [76].

Interestingly, Saheb Sharif-Askari et al. [37] determined that gene expression levels of MPO were significantly upregulated in saliva and blood from severe compared with asymptomatic COVID-19 patients. These findings suggest that the MPO expression in saliva could be used as a non-invasive marker for COVID-19 severity. Severe COVID-19 is strictly related to innate immune dysregulation, an elevated neutrophil-to-lymphocyte ratio, and cytokine storm [77,78]. These mechanisms associated with SARS-CoV-2 infection provoke oxidative stress, leading to lung tissue damage [79]. In severe COVID-19, increased MPO activity causes soluble endothelial glycocalyx (EG) shedding and its inhibition protects against EG degradation [80].

### 4.6. Autoimmunological Disorders

Based on immune complexome analysis, Yamane et al. [40] identified MPO as new IC-antigens that were frequently and specifically detected in the saliva of patients with Sjögren’s syndrome (SS). The authors suggest that MPO as a neutrophil intracellular protein indicates that repeated neutrophil destruction caused by altered autoimmunity could be involved in the pathogenesis of SS. In patients with ANCA-associated vasculitis in primary SS course, most cases present anti-MPO specificity [81,82].

In rheumatoid arthritis (RA), rheumatoid factor as an immune complex inappropriately activates neutrophils, affecting their longevity and function [83]. After release from degranulating neutrophils, MPO produces oxidants which activate proMMPs and inactivate tissue inhibitor of metalloproteinases 1, leading to inflammatory and oxidative damage in the joint tissues [84]. Moreover, MPO and neutrophil elastase, significantly increased in serum and synovial fluid in RA patients, can enhance the destructive MMP cascade [85,86].

The study by Yilmaz et al. [41] showed that only serum levels of MPO were significantly elevated in patients with RA compared with the healthy controls, regardless of periodontal status. In turn, salivary MPO concentrations were increased but without statistical significance. For saliva, MPO levels differ significantly between systemically healthy periodontitis patients and control subjects.

### 4.7. Other Disorders

Akcalı et al. [16] found no significant differences in salivary myeloperoxidase levels between patients with polycystic ovary syndrome (PCOS) and healthy subjects, regardless of gingival inflammation. Significantly higher serum myeloperoxidase levels were observed in PCOS patients with gingivitis than generally healthy individuals with gingivitis. Also, PCOS patients exhibited a positive correlation between salivary MPO levels and clinical periodontal parameters. Due to the low-grade chronic inflammation in PCOS, elevated local and systemic proinflammatory cytokines stimulate the production of MMP-9 and MPO, initiating the proteolytic cascades [87]. Also, oxidative imbalance plays a role in the pathogenesis of PCOS, e.g., the MPO G-463A variant is related to a higher risk of PCOS [88]. The co-presence of insulin resistance is associated with increased MPO activity and ROS production, potentiating leukocyte-endothelium interactions [89].

Increased MPO promotes the degradation of toxic lysosomal deposits. However, chronically elevated MPO activity causes lysosomal stress and cell death [90]. In the study by Drążewski et al. [19], patients with Pompe disease had significantly increased MPO levels compared to patients with mannosidosis. Patients with lysosomal storage diseases did not appear to differ significantly from healthy controls.

Furthermore, Dodds et al. [18] showed that MPO concentrations in stimulated parotid saliva were nearly four-fold higher in patients with type 2 diabetes mellitus (T2DM) than in control subjects. In the pathogenesis of T2DM, a ROS flux is an independent factor modulated by MPO, regardless of metformin therapy and concomitant cardiovascular diseases [91]. Elevated MPO activity is responsible for endothelial dysfunction and atherosclerosis, leading to T2DM vascular complications [92]. The specific variant of MPO gene contributes to the higher predisposition for T2DM and its vascular complications, suggesting MPO as a probable therapeutic target for T2DM [93]. Interestingly, MPO is related to insulin resistance and inflammation in overweight individuals with first-degree relatives suffering from T2DM, increasing the risk of developing this disease in these subjects [94]. 

### 4.8. Study Limitations

Among the limitations of the review are the heterogeneity of the included studies in terms of systemic disease diagnoses, laboratory methods for determining MPO levels (as concentrations or activity), and considered sample sizes, associated directly with the wide time range of published results (from 1994 to 2023). For this reason, it is not possible to compare the findings between the different studies presented. 

The most common methodological bias sources included the missing data on blinding, randomisation, and justification for the sample size. Also, in most studies, statistical analysis was limited to comparisons of MPO levels, without assessing predictive values, e.g., by analysing ROC curves.

Moreover, MPO concentrations were determined in a large part of the studies, but activity was only determined in two studies. It should be stressed that the level of antioxidants in saliva can be influenced by individual factors, such as smoking, age, gender, or oral health (especially periodontal status). The promising introduction of non-invasive saliva collection into daily clinical diagnostics must overcome barriers such as the standardisation of conditions related to the processing (e.g., collection, storing, or processing duration and temperature). These factors are not insignificant for the MPO outcomes achieved by the different researchers. Thus, it is impossible to critically compare the results between studies.

In addition, we did not include studies reported in conference proceedings and other grey literature that might affect the finding of this systematic review. In turn, we discussed one of our most recent research projects, which has not yet been published, but expands on previous observations. Once the presence of MPO alterations in saliva has been potentially established, it would be useful to further investigate whether salivary MPO could be a reliable biomarker compared to serum MPO, which was not within the scope of the current review. However, a similar heterogeneity of studies can be expected, making it impossible to carry out any meta-analysis again.

## 5. Conclusions

According to our systematic review, myeloperoxidase could be altered in the saliva of patients with systematic diseases, especially cardiovascular or gastrointestinal diseases. However, further research is necessary to validate these findings.

## Figures and Tables

**Table 1:** Inclusion and exclusion criteria according to the PI(E)COS.

Parameter	Inclusion Criteria	Exclusion Criteria
Population	Patients aged from 0 to 99 years, both genders	
Intervention/Exposure	systemic diseases (e.g., cardiovascular diseases, gastrointestinal diseases)	other diseases (e.g., oral diseases)
Comparison	not applicable	
Outcomes	salivary levels of myeloperoxidase	serum levels of myeloperoxidase, salivary levels of other antioxidants
Study design	case-control, cohort, and cross-sectional studies	literature reviews, case reports, expert opinion, letters to the editor, conference reports
	published until 5 May 2023	not published in English

**Table 2:** General characteristics of included studies.

Author, Year	Setting	Study Group (F/M), Age	Control Group (F/M), Age	Diagnosis	Inclusion Criteria	Exclusion Criteria	Smoking Status
Akcalı et al., 2017 [16]	Turkey	80 (80/0): 45 periodontally healthy, 35 with gingivitis; NR	45 (45/0): 25 periodontally healthy, 20 with gingivitis; NR	Polycystic ovary syndrome (PCOS)	PCOS diagnosed based on Rotterdam criteria	Hyperandrogenism, diabetes mellitus, hyperprolactemia, congenital adrenal hyperplasia, thyroid disorders, Cushing’s syndrome, hypertension, hepatic or renal dysfunction, BMI > 30 kg/m^2^, and cardiovascular diseases, medication such as oral contraceptive agents, steroid hormones, insulin-sensitizing drugs and antibiotics or anti-inflammatory drugs that could affect their periodontal status during the last 6 months prior to the study, smoking	No smokers
Akpinar et al., 2012 [17]	Turkey	32 (8/24); 43.79 ± 12.72	24 (8/16); 44.7 ± 13.75	Obstructive sleep apnea (OSA)	Patients with complaints of snoring, excessive daytime sleepiness, and apnea, whereas control subjects had no distinct complaints	Oropharyngeal and dental infections, ongoing dental treatments, periodontal and gingival inflammation, laryngeal pathologies, a history of asthma, chronic bronchitis, allergic rhinitis, chronic obstructive pulmonary disease, and acute airway infection, smoking, a history of continuous positive airway pressure use and upper airway surgery for OSA, including nasal surgery	No smokers
Dodds et al., 2000 [18]	USA	233 (113/120) DM, mean 61.2/63.9; 227 (136/91) hypertension, 64.7/62.2	240 (106/134), mean 55.3/55.9	Diabetes mellitus (DM) type 2, hypertension	DM identified by modified WHO criteria or currently taking diabetic medications; hypertension classified by diastolic blood pressure ±95 mm Hg or currently taking anti-hypertensive medications	NR	NR
Drążewski et al., 2017 [19]	Poland	36 (13/23), range: 7–30	NA	Lysosomal storage diseases (LSDs)	Patients participated in rehabilitation training courses in the SMRD center	NR	NR
Floriano et al., 2009 [20]	USA	56 (36/20), mean 54.8	59 (34/25), mean 49.3	Acute myocardial infarction (AMI)	Patients within 48 h of onset of symptoms of AMI, at least 18 years old	Fever, stroke, immune disorders, use of steroidal medications, organ complications/failure, and inability to provide saliva	NR
Foley et al., 2012 [21]	USA	19 (12/7), 58.58 ± 13.41	97 (59/38), 48.6 ± 8.6	Hypertrophic cardiomyopathy (HCM)	Underwent alcohol septal ablation as treatment for HCM	Age < 18 years, unable or unwilling to provide informed consent or provide samples, recently treated with chemotherapeutic drugs, anti-organ rejection drugs, or significant immune modulators within the past 3 months or during the course of the study, febrile illness, or active infection at the time of enrollment, pregnancy	Current smokers (n = 9)
Janšáková et al., 2021 [22]	United Kingdom	27 (17/10) OFG, 38.76 ± 14.53; 29 (10/19) CD, 37.13 ± 11.78; 14 (3/11) CD + OFG, 44.5 ± 15.1	22 (10/12), 35.09 ± 10.12	Crohn’s disease (CD), orofacial granulomatosis (OFG)	In a remission phase of the disease; no acute complications or presenting of co-morbidities at the date of the collection	Presence of another systemic disease, oral diseases, e.g., periodontitis, oral lichen planus, acute illnesses, e.g., cold, gastroenteritis, infection of urogenital tract and taking anticholinergic drugs or treatment affecting salivary flow, pregnancy, age under 18 years	Smokers included (NR)
Johansson et al., 1994 [23]	India	34 (NR), range: 8–12	34 (sex- and age-matched)	Chronic protein-energy malnutrition (PEM)	Selected from 8- to 12-year-old pupils attending St. Mary’s School, Madras, India; with a defined date of birth and without other conditions or any known disease at the time of examination	NR	NA
Karolewska et al., 2008 [24]	Poland	44 (19/25), mean 10.26, range: 3–17	23 (NR), mean 8.7, range: 5–14	Leukemia	Children with newly diagnosed acute leukemia, able to participate in saliva collection and whose parents signed the informed consent	Children younger than 3 years	NA
Kirstilä et al., 1994 [25]	Finland	15 (6/9), range: 7–67	15 (6/9), range: 7–63	Common Variable Immunodeficiency (CVI)	Diagnosed for CVI in Turku University Central Hospital	NR	NR
Lahdentausta et al., 2018 [26]	Finland	163 (45/118), mean 63.0 ± 9.5	290 (102/188), mean 64.1 ± 8.7	Acute coronary syndrome (ACS)	≥50% stenosis in at least one coronary artery, episode of typical chest pain for ischemia and elevated cardiac enzymes.	“ACS-like, no significant CAD” (including takotsubo patients)	Smokers (*n* = 94)
Lenander-Lumikari et al., 1998 [27]	Finland	26 (21/5), range: 25–50	33 (23/10), range: 25–50	Asthma	Age ranging from 25 to 50 years and diagnosed asthma	Medication for psychiatric diseases, diabetes or any other disease that may directly or indirectly affect the oral cavity	Smokers (*n* = 5)
Lenander-Lumikari et al., 2000 [28]	Finland	128 (104/24), mean 42.7 ± 14.7	55 (41/14), mean 39.1 ± 12.7	Coeliac disease	Invited members of the Coeliac Association of Turku, Finland, without any discrimination based on age or sex, followed a strict gluten-free diet	Diagnosis based only on positive serological tests	NR
Mahmood et al., 2019 [29]	Sweden	23 (5/18), range: 60–69	NA	Coronary artery disease (CAD)	Recent coronary event, i.e., acute coronary syndrome and/or revascularization with either percutaneous coronary intervention or coronary artery bypass grafting	Age > 75 years, severe heart failure, neoplastic disease, major clinical depression, chronic liver and renal failure, chronic immunologic disorders or treatment with immunosuppressive/anti-inflammatory agents, serious physical or psychological diseases interfering with performing an exercise test, and inability to understand the Swedish language	Smokers (*n* = 5)
Mellanen et al., 1996 [30]	Finland	56 (10/46), mean 37.7, range: 23–68	10 (5/5), mean 36, range: 27–58	Human immunodeficiency virus (HIV)	Voluntary HIV-seropositive patients visiting the Aurora Hospital Dental Clinic, Helsinki, Finland	NR	NR
Nijakowski et al., 2021 [31]	Poland	27 (10/17) CD, range: 28–48; 24 (7/17) UC, range: 24–40.5	51 (17/34), range: 26–40	Crohn’s disease (CD), ulcerative colitis (UC)	Adult patients of both sexes, with inflammatory bowel diseases, qualified for biologic treatment	Concomitant autoimmune diseases (including diabetes), periodontal disease or other overt inflammatory lesions in the oral cavity, taking medications known to affect salivation	Smokers (CD *n* = 7, UC *n* = 1)
Nijakowski et al., 2021 [32]	Poland	27 (10/17) CD, range: 28–48; 24 (7/17) UC, range: 24–40.5	NA	Crohn’s disease (CD), ulcerative colitis (UC)	Adult patients of both sexes, with inflammatory bowel diseases, qualified for biologic treatment - patients with active, moderate to severe disease, not responding to previous conventional full-dose therapy or showing intolerance to such therapy (e.g., allergic reactions)	Concomitant autoimmune diseases (including diabetes), periodontal disease or other overt inflammatory lesions in the oral cavity, taking medications known to affect salivation	Smokers (CD *n* = 7, UC *n* = 1)
Nizam et al., 2015 [33]	Turkey	37 (12/25): 17 mild-to-moderate (8/9), range: 29–64; 20 severe (4/16), range: 26–61	13 (8/5), range: 21–59	Obstructive sleep apnea (OSA)	Complaints of sleep apnea-related symptoms	Immunological disorders, diabetes mellitus, received antibiotic treatment within the last 3 months, and periodontal treatment within the last 6 months, or had less than 20 teeth and wearing removable dentures	Smokers (*n* = 8), ex-smokers (*n* = 6)
Palm et al., 2014 [34]	Germany	98 (45/53), mean 68.2 ± 9.7	100 (47/53), mean 69.1 ± 5.2	Acute ischemic stroke	Patients with a first-ever ischemic stroke between the age of 18 and 80 years	Clinical or laboratory signs of acute infection at time of stroke onset	Smokers (*n* = 28), ex-smokers (*n* = 58)
Polizzi et al., 2020 [35]	Italy	62 (32/30): 31 periodontally healthy (17/14), range: 46–58; 31 with periodontitis (15/16), range: 47–56	62 (31/31): 31 periodontally healthy (16/15), range: 48–56; 31 with periodontitis (15/16), range: 47–57	Cardiovascular disease (CVD)	At least ≥18 years; a diagnosis of CVD with ≥50% of stenosis of at least one coronary artery verified by coronary angiography, or past or current percutaneous coronary intervention	Contraceptive drugs, antibiotics, anti-inflammatory or immunosuppressive drugs during the three months previous the trial; gestation or suction; intake of alcohol; anesthetic allergy; intake of nifedipine, hydantoin or cyclosporin; any type of periodontal treatment in the three months before baseline	Smokers (*n* = 4), ex-smokers (*n* = 4)
Rathnayake et al., 2015 [36]	Sweden	200 (32/168), mean 61 ± 8	200 (32/168), mean 61 ± 8	Coronary Artery Disease (CAD)	<75 years old and admitted to a coronary care unit with a first myocardial infarction	Undergone cardiac valvular surgery or had language barriers preventing them to complete study procedures	Smokers (*n* = 19), ex-smokers (*n* = 103)
Saheb Sharif-Askari et al., 2021 [37]	United Arab Emirates	7 asymptomatic (NR), mean 44 ± 6; 10 severe (NR), mean 53 ± 11	5 (NR), mean 34 ± 8	SARS-CoV-2 infection	COVID-19 patients with different disease severity	NR	NR
Salvador et al., 2017 [38]	Brasil	27 (12/15), mean 42 ± 17.8	24 (13/11), mean 41 ± 14.9	Undergoing hematopoietic stem cell transplantation (HSCT)	At least 14 years of age, scheduled for autologous or allogeneic HSCT and planned treatment consisting of a myeloablative conditioning regimen with high-dose chemotherapy, without radiotherapy; intact oral mucosal lining, no infectious foci or other associated pathologies.	Patients who needed tracheal intubation, or presented mental confusion, or died	NR
van Leeuwen et al., 2018 [39]	The Netherlands	20: part A: 12 (5/7), range: 43–68; part B: 8 (3/5), range: 53–67	NA	Multiple myeloma (MM)	Adult MM patients undergoing HDM (200 mg/m^2^, infused during 1 h) and autologous HSCT	Patients who did not understand the Dutch language	NR
Yamane et al., 2021 [40]	Japan	9 (8/1), range: 38–77	7 (7/0), range: 26–93	Sjögren’s syndrome (SS)	Patients who met the internationally accepted 2002 American–European Consensus Group Sjögren’s syndrome classification criteria	NR	NR
Yilmaz et al., 2023 [41]	Finland	49 (36/13): 26 with periodontitis (18/8), range: 33–68; 23 periodontally healthy (18/5), range: 40–68	48 (26/22): 24 periodontally healthy (14/10), range: 34–66; 24 with periodontitis (12/12), range: 35–63	Rheumatoid arthritis (RA)	RA patients undergoing treatments and regular follow-ups	Having <16 teeth, periodontal treatment history and antibiotic use within at least 3 months or more before the initiation of the study, inflammatory and/or mucocutaneous disease and disorders of the oral cavity, additional general disorders or diseases such as diabetes mellitus, renal, hepatic disorders, or HIV as well as pregnancy and lactating period, a history of transplantation, diagnosed with other forms of arthritis, recent quitters (<2 years), and occasional smokers	Smokers included (NR)

Legend: F, female; M, male; NA, not applicable; NR, not reported; USA, the United States of America.

**Table 3:** Detailed characteristics of included studies considering methods of collection and analysis of saliva.

Study	Type of Saliva and Method of Collection	Centrifugation and Storing	Method of MPO Determination	Salivary Biomarkers
Akcalı et al., 2017 [16]	Unstimulated whole saliva into sterile 50 mL tubes for 5 min	Placed on ice, supplemented with EDTA-free Protease Inhibitor Cocktail, centrifuged at 10,000× *g* for 15 min at 4 °C, stored at −80 °C until analysis	ELISA	MPO, MMP-9, NE, TIMP-1
Akpinar et al., 2012 [17]	Unstimulated whole saliva 2–3 mL for 5 min	NR	Flow cytometry using a fluorescent bead immunoassay method	MPO
Dodds et al., 2000 [18]	Unstimulated whole saliva for 5 min, stimulated parotid for 5 min using a modified Carlson–Crittenden cup, and submandibular/sublingual saliva stimulated for 3 min and unstimulated for 5 min using a micropipette connected to a mini-vacuum pump; stimulation by swabbing the lateral surfaces of the tongue with 0.1 mol/L citric acid every 30 s	The volume determined gravimetrically, stored at −80 °C	ELISA	MPO, salivary peroxidase, cystatin, albumin, lactoferrin, lysozyme, secretory IgA
Drążewski et al., 2017 [19]	Unstimulated whole saliva into a sterile container	Centrifuged and stored at −80 °C until analysis	ELISA	MPO, TAS, MCP-1, TNF-R1 and TNF-R2, VEGF, sICAM-1, MMP-2
Floriano et al., 2009 [20]	Unstimulated whole saliva	Transported on ice, centrifuged, stored at –80 °C until analysis	Luminex® IS100-based multiplex kits	MPO, CRP, MMP-9, IL-1β, slCAM-1, adiponectin, MCP-1, Gro-α, E-selectin, IL-18, ENA-78, sVCAM-1, MYO, CK-MB, cTnl, BNP, Fractalkine, RANTES, IL-6, sCD40-L, TNF-α
Foley et al., 2012 [21]	Unstimulated whole saliva 5 mL at baseline and at 8, 16, 24, and 48 h post-procedure; according to a modification in the method described by Navazesh	Immediately placed on ice, transported within 10 min, centrifuged, stored at –80 °C until analysis	Luminex^®^ IS100-based multiplex kits	MPO, MMP-9, CK-MB, MYO, cTnI, BNP, CRP
Janšáková et al., 2021 [22]	Unstimulated whole saliva by passive drooling for 10 min into sterile tubes	Kept on ice, centrifuged at 7000× *g* for 5 min at 4 °C, stored at –20 °C until analysis	ELISA	MPO, IgA, lactoferrin
Johansson et al., 1994 [23]	Whole saliva: unstimulated by drooling for 10 min and paraffin-stimulated expectorated for 5 min into ice-chilled, graded test tubes	Centrifuged at 14,500× *g* for 15 min at 4 °C, stored at –20 °C	Non-isotopic immunometric assays with biotin-labeled antibodies and avidin alkaline phosphatase label	Unstimulated: MPO, thiocyanate, lactoferrin, lysozyme, BAGP, total IgG and IgA, specific anti-S. mutans IgA; stimulated: hexosamines, fucose, sialic acid, amylase, sodium, potassium, calcium, chloride
Karolewska et al., 2008 [24]	Unstimulated whole saliva into plastic test tubes placed in a flask of ice	Centrifuged at 3000× *g* for 15 min, stored at –30 °C until analysis	The modified method by Mansson-Rahemtulla et al., based on the time of oxidation of Cl– to OCl– present in the substrate Nbs-Cl	MPO, salivary peroxidase, lysozyme, lactoferrin, secretory IgA
Kirstilä et al., 1994 [25]	Paraffin-stimulated whole saliva	Centrifuged at 18,000× *g* for 10 min at 4 °C, stored at −20 °C until analysis	Non-isotopic immunometric assays with biotin-labeled antibodies and avidin alkaline phosphatase label	MPO, lysozyme, lactoferrin, salivary peroxidase, hypothiocyanite, thiocyanate, IgA, IgG, and IgM (total and specific anti-S. mutans)
Lahdentausta et al., 2018 [26]	Paraffin-stimulated whole saliva for 5 min and at least 2 mL by expectoration	Centrifuged at 9300× *g* for 3 min and used for the analysis	ELISA	MPO, MMP-8, MMP-9, TIMP-1
Lenander-Lumikari et al., 1998 [27]	Paraffin-stimulated whole saliva into chilled, graduated glass tubes for 5 min	Centrifuged at 12,000× *g* for 10 min at 4 °C, stored at −20 °C until analysis	Non-isotopic immunometric assays with biotin-labeled antibodies and avidin alkaline phosphatase label	MPO, lactoferrin, lysozyme, salivary peroxidase, calcium, potassium, sodium, thiocyanate
Lenander-Lumikari et al., 2000 [28]	Paraffin-stimulated whole saliva into chilled, graduated glass tubes for 5 min	Immediately centrifuged at 16,000× *g* for 10 min at 4 °C, stored at −20 °C until analysis, analyzed within 2 months	The modified method by Mansson-Rahemtulla et al., based on the time of oxidation of Cl– to OCl– present in the substrate Nbs-Cl	MPO, salivary peroxidase, IgA, IgG and IgM, amylase, total protein
Mahmood et al., 2019 [29]	Unstimulated whole saliva with oral cotton swabs placed under the tongue for 2 min; prior to the bicycle test, directly after, and 30 min after the test completion	Immediately placed on ice, centrifuged 10,000× *g* for 5 min, stored at −70 °C until analysis	Magnetic bead-based luminex assay	MPO, MMP-9
Mellanen et al., 1996 [30]	Paraffin-stimulated whole saliva about 5 mL of whole paraffin stimulated saliva	Immediately frozen and stored at −20 °C until analysis	Quantitative dot blotting	MPO, MMP-1, MMP-3, MMP-8
Nijakowski et al., 2021 [31]	Unstimulated whole saliva by passive drooling up to 20 min, as described by Navazesh	Centrifuged and stored at −80 °C until analysis	ELISA	MPO, catalase, TNF-R1, serpin E1/PAI-1, S100A8/calprotectin, IgA
Nijakowski et al., 2021 [32]	Unstimulated whole saliva by passive drooling up to 20 min, as described by Navazesh	Centrifuged and stored at −80 °C until analysis	ELISA	MPO, IgA
Nizam et al., 2015 [33]	Unstimulated whole saliva expectorated into polypropylene tubes	Centrifuged at 800× *g* for 10 min at room temperature	ELISA	MPO, MMP-2, MMP-8, MMP-9, TIMP-1, NE, NGAL
Palm et al., 2014 [34]	Paraffin-stimulated whole saliva for 5 min and at least 2 mL by expectoration	Stored at −70 °C until analysis	ELISA	MMP-8, MPO, TIMP-1, IL-1β
Polizzi et al., 2020 [35]	Stimulated whole saliva by chewing a cotton roll for 2 min using the salivette method	Centrifuged at 1000× *g* for 2 min at 4 °C, stored at −20 °C	Magnetic bead-based luminex assay	MPO
Rathnayake et al., 2015 [36]	Paraffin-stimulated whole saliva up to 10 min into a graded test-tube until 2 mL	Centrifuged at 500× *g* for 5 min at 5 °C, stored at −80 °C until analysis	ELISA	MMP-8, MMP-9, MPO, TIMP-1
Saheb Sharif-Askari et al., 2021 [37]	Unstimulated whole saliva	NR	qRT-PCR	125 oxidative stress genes: 37 pro-oxidant genes, 32 oxidative-responsive genes, and 56 antioxidant genes (including MPO)
Salvador et al., 2017 [38]	Unstimulated whole saliva into sterile tubes for 5 min at three different moments of treatment	Centrifuged at 1500 rpm for 10 min, stored at −80 °C until analysis	ELISA	MPO, CXCL8/IL-8, nitrite
van Leeuwen et al., 2018 [39]	Whole saliva: unstimulated for 5 min and paraffin-stimulated for 1 min into previously weighed plastic cups	Centrifuged at 10,000 rpm for 5 min, stored at −80 °C until analysis	ELISA	MPO, mucin 5B, albumin, total IgA, lactoferrin
Yamane et al., 2021 [40]	Stimulated whole saliva using the Saxon test	NR	Immune complexome analysis	MPO, NE, neutrophil defensin 1, small proline-rich protein 2D cathepsin G, nuclear mitotic apparatus 1, phosphatidylinositol 4-phosphate 3-kinase C2 domain-containing subunit gamma
Yilmaz et al., 2023 [41]	Unstimulated whole saliva samples into calibrated 2 mL plastic tubes for 5 min	Centrifuged at 6000× *g* for 5 min, stored at −80 °C until transferred	ELISA	active-MMP-8, TIMP-1, NE

Legend: BAGP, bacteria-agglutinating protein; BNP, brain natriuretic peptide; cTn, cardiac troponin; CK-MB, creatine kinase-MB; CRP, C-reactive protein; EDTA, ethylenediaminetetraacetic acid; ELISA, enzyme-linked immunosorbent assay; ENA-78, epithelial cell-derived neutrophil-activating peptide 78; Gro-α, growth related protein-α; Ig, immunoglobulin; IL, interleukin; MCP-1, monocyte chemoattractant protein-1; MMP, matrix metalloproteinase; MPO, myeloperoxidase; MYO, myoglobin; NE, neutrophil elastase; NGAL, neutrophil gelatinase-associated lipocalin; NR, not reported; qRT-PCR, quantitative reverse transcriptase polymerase chain reaction; PAI-1, plasminogen activator inhibitor-1; RANTES, regulated on activation, normal T expressed and secreted; sCD40L, soluble cluster of differentiation ligand; sICAM-1, soluble intercellular adhesion molecule-1; sVCAM-1, soluble vascularization cellular adhesion molecule-1; TAS, total antioxidant status; TIMP-1, tissue inhibitor of matrix metalloproteinase-1; TNF-R, tumor necrosis factor receptor; VEGF, vascular endothelial growth factor.

**Table 4:** Reported statistically significant differences in salivary MPO levels between patients with systemic diseases and healthy subjects.

Study	Diagnosis	Concentration/Activity	Study Group	Control Group	*p*-Value	AUC
Akpinar et al., 2012 [17]	Obstructive sleep apnoea	ng/mL	6.40 ± 2.82	3.92 ± 0.34	<0.0001	-
Dodds et al., 2000 [18]	Diabetes mellitus	µg/mL	0.81 ± 0.10	0.21 ± 0.06	0.0117	-
Floriano et al., 2009 [20]	Acute myocardial infarction	-	Me_study_/Me_control_ 1.91		-	0.71
Karolewska et al., 2008 [24]	Leukemia	IU/mg TP	0.21 ± 0.16	0.27 ± 0.16	0.013	-
Lenander-Lumikari et al., 1998 [27]	Asthma	ng/mL	164.4 ± 136.5	95.2 ± 110.0	<0.05	-
Lenander-Lumikari et al., 2000 [28]	Coeliac disease	mU	0.61 ± 0.29	0.36 ± 0.12	≤0.001	-
Nijakowski et al., 2021 [31]	Crohn’s disease	ng/µg TP	0.167 (0.046–0.577)	0.239 (0.070–0.685)	<0.001	CD vs. UC, ng/mL
	Ulcerative colitis		0.055 (0.011–0.124)			0.69
Palm et al., 2014 [34]	Ischemic stroke	ng/mL	Me (IQR) 999.55 (1248.56)	Me (IQR) 2092.98 (3894.23)	<0.0001	-
Polizzi et al., 2020 [35]	Cardiovascular disease	ng/mL	83.2 (77.4–101.5)	-	<0.01	-
Rathnayake et al., 2015 [36]	Coronary artery disease	ng/mL	1637 ± 1386	1899 ± 1447	0.02	-

Legend: -, not reported; TP, total protein; AUC, area under curve; CD, Crohn’s disease; UC, ulcerative colitis.

## Data Availability

Data are available on request from the corresponding author.
